# Loading conditions in the spine, hip and knee during different executions of back extension exercises

**DOI:** 10.1186/s13102-017-0074-0

**Published:** 2017-04-24

**Authors:** Florian Schellenberg, Nicole Schmid, Ramona Häberle, Nicole Hörterer, William R. Taylor, Silvio Lorenzetti

**Affiliations:** Institute for Biomechanics, ETH Zürich, HCP H 21.1, Leopold-Ruzicka-Weg 4, 8093 Zürich, Switzerland

**Keywords:** Strength training, External joint moments, Ranges of motion, EMG, Trunk, Lower extremities, Rehabilitation

## Abstract

**Background:**

Back extension (BE) is a strength exercise for training the dorsal trunk and hip muscles. To optimise training recommendations that avoid overloading and possible injury, the aim of this study was to determine the loading conditions and the influence of different execution forms of BE on spine, hip and knee ranges of motion (RoMs), joint moments and muscle activity.

**Methods:**

The kinematics, kinetics and muscle activity (EMG) of two execution types (BE_h_: dynamic hip, BE_s_: dynamic spine) and two versions (one-legged and two-legged) of BE were measured in 16 subjects. RoMs and external joint moments were calculated using an inverse dynamics approach and analysed with a linear mixed model.

**Results:**

Although lumbar spine flexion was observed in both execution types, thoracic spine flexion predominantly occurred during BE_s_, whereas thoracic spine extension was observed during BE_h_. Larger maximal back and hip moments were observed for BE_h_ than for BE_s_. The activity of the dorsal back and hip muscles, as observed using EMG, was increased for one-legged executions.

**Conclusion:**

To strengthen the hips and lower back, BE_h_ seem to be more efficient due to the higher moments, with higher or similar RoMs in the hip and lower back. One-legged BE_s_ seem to provide an effective training for the hamstrings and hip regions without subjecting the spine to excessive loading, possibly promoting this as an effective exercise during training and rehabilitation.

## Background

Strength exercises are part of most training and rehabilitation programs. The back extension (BE) is often performed by athletes as well as low back pain patients to strengthen the dorsal trunk and hip musculature [1]. Because a majority of injuries during strength training occur due to the overloading and incorrect execution of the exercises [2], biomechanical knowledge of the loading conditions that occur during BE exercises, especially in the hip and knee joints and in the lower back, is important to provide safe and efficient recommendations for training and rehabilitation.

Two dynamic types of BE are commonly used: the activity can be predominantly driven either by movement at the hip while the spine is stabilised (BE_h_) or by movement at the spine executed with a stabilised hip (BE_s_). In addition, one- and two-legged versions are also possible and frequently used. Historically, the execution of BE_s_ has been based on the Sorensen test, which was developed to assess the isometric endurance of the dorsal trunk muscles [3, 4]. During this test, subjects lie on a table with their held legs horizontal to the ground and attempt to hold their unsupported upper body stable for as long as possible. In a more recent development of the exercise, instead of a horizontal table, a variable-angle Roman chair is used, which allows the legs to be placed at an angle to the horizontal of, e.g., 45° [1, 5–8], and thereby allows the highest loading conditions (i.e., when the upper body is horizontal) to act at different hip flexion angles [9]. This idea has been supported by electromyography (EMG) measurements that demonstrated changes in lumbar muscle activity resulting from different starting postures and the associated changes in muscle lengths [10]. In addition, higher endurance of the lumbar dorsal trunk muscles has been observed when BE was performed on a variable-angle Roman chair compared to the horizontal Sorensen set-up [5]. These authors assumed that the contribution of the passive structures, mainly the thoraco-lumbar fascia, was greater due to the increased stretch of the hip extensors in the variable-angle Roman chair set-up and that less lumbar muscle effort was thus required to support the mass of the upper body.

Many studies have investigated the muscle recruitment of trunk and hip muscles in different forms of BE exercises. Direct comparisons between these studies should be considered with caution because the test protocols and analyses were different. Lumbar and thoracic trunk muscles were highly activated during BE without additional loading (39–56 and 43% maximum voluntary contraction (MVC) on a 40° Roman chair) and similar between different execution forms. Gluteal and hamstring activities can be slightly increased using a more horizontal BE execution (40° Roman chair: 15–23%, 12–16% MVC, respectively; horizontal position: 22–23 and 16-24% MVC, respectively) [1, 11].

Changing the kinematic parameters during BE lead to different activation levels and different spine postures (e.g., +18% activation level for lumbar extensors with an accentuated lumbar lordosis of +25% by performing BE with an internal rotated hip) [8]. Therefore, different loading conditions act on the spine with different execution forms and lead to different risks of injury because disk deformation and ligament and spinal loading can be reduced if BE exercises are performed with neutral lordosis [11].

Distinct recommendations are missing in the literature and controversially discussed, such as for the specific strengthening of the lumbar trunk muscles relative to hip extensors. Whereas Da Silva and co-workers [1] stated that a 40° Roman chair reduced the activity of the biceps femoris but did not alter the activity of the lumbar back extensors compared to a horizontal position, Mayer and co-workers [9] found that the lumbar back extensors were more active in a more horizontal position. Furthermore, Larivière and co-workers [7] stated that the 40° condition was not well suited to specifically fatigue the dorsal back muscles relative to the dorsal hip muscles. Moreover, increased external load or several sets of exercises could increase the muscle activity of the hip extensors to a higher degree than that of the lumbar back extensors and therefore more specifically train the dorsal hip muscles [12]. In contrast, with an external load of 60% of 1RM, De Ridder and co-workers [13] stated that the gluteal muscles played a smaller role compared to the lumbar muscles. Regarding the breathing pattern during strength training, the National Strength and Conditioning Association recommends to instruct athletes to exhale through the sticking point and to inhale during the less stressful phase of the repetition [14].

A detailed biomechanical analysis of the movement, loading patterns and muscle activities of the BE exercise including trunk, hip and knee joints, is missing. However, as stated above, to avoid injuries and derive training recommendations, knowledge of the loading conditions is fundamental.

Therefore, the aim of this study was to determine the influence of different execution forms of BE exercises on the spine, hip and knee ranges of motion (RoMs) and the corresponding external joint moments as well as muscle activities. The different execution forms include single or double legged execution, performed either using an isometric hip and a dynamic spine or using a dynamic hip and an isometric spine.

## Methods

### Participants

Sixteen subjects (8male, 8female, age 26.3 ± 4.2 years, body mass 71.9 ± 15.1 kg, height 1.76 ± 0.09 m) were evaluated and provided written informed consent to participate in this study. They were required to have personal experience in strength training, be physically active at least three hours per week and have neither past surgery on back, hip or knee nor any current injuries or illnesses. All subjects were instructed to wear normal sports shoes and shorts, and female subjects additionally wore a bikini top. The study was approved by the ethics committee of ETH Zurich, Switzerland (EK 2014-N-31).

### Data acquisition

To analyse the kinematic motion of the body, 21 cameras (MX40 and MX160) of an opto-electronic motion system (Vicon, Oxford Metrics Group, Oxford, UK) operating at a sampling frequency of 100 Hz were used. 77 reflective markers were placed onto the subjects according to the method described by List and co-workers [15]. The markers were attached to the upper and lower extremities, the pelvis, and the trunk using double-sided, skin-friendly tape. The markers on the feet and spine had a diameter of 9 mm, whereas the markers on the other segments had a diameter of 14 mm. To functionally determine the joint centres of the ankle, knee and hip joints, the subjects performed standardised basic motion tasks [15].

**Fig. 1:**
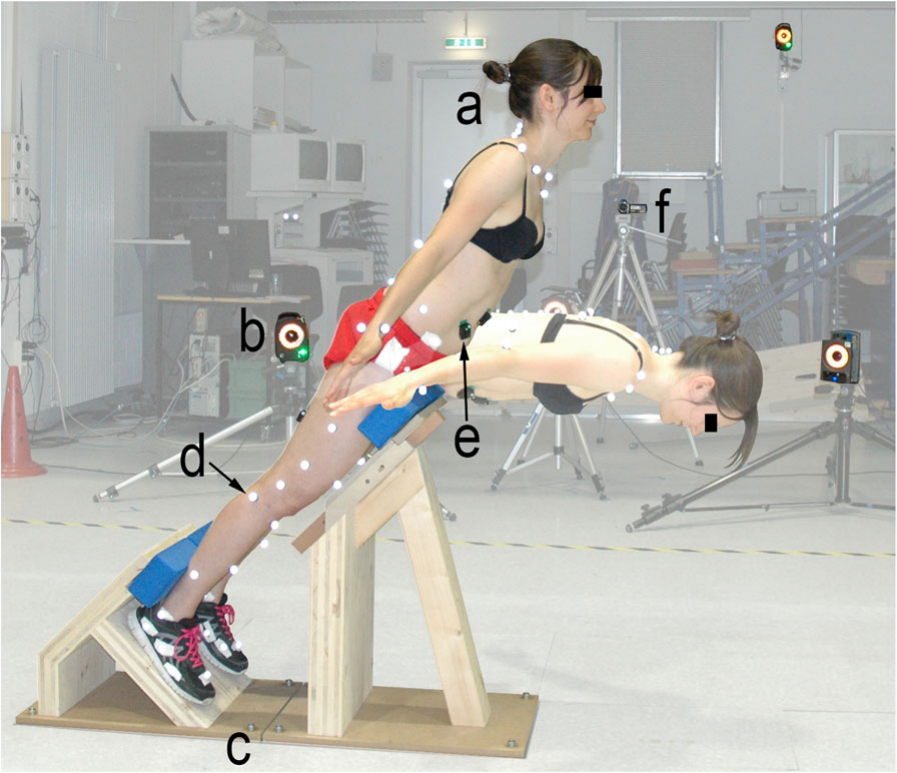
Measurement Setup: Back Extension (BE) exercises on a 45° BE bench: a: subject, b: opto-electronic motion cameras, c: two decoupled force plates, d: reflective markers, e: EMG sensors, f: video camera

The ground reaction forces were measured using two 40 × 60 cm^2^ force plates (type 9281B Kistler Instrumente AG, Winterthur, Switzerland) operating at 2 kHz. The specially constructed BE bench consisting of two mechanically decoupled parts was mounted onto these force plates (Fig. 1). The frontal part was adjusted in height such that the subject’s hip joint centre was just above the edge of the bench. Moreover, the force plates were specifically calibrated to correct the centre of pressure [16].

To record muscle activities, surface EMG was used. 16 EMG sensors (Trigno^™^ Wireless EMG System, Delsys, USA) operating at a frequency of 2 kHz were placed bilaterally onto the following muscle bellies [17]: *M. gluteus maximus* (GlutMax), *M. gluteus medius* (GlutMed), lateral hamstrings (HamLat), medial hamstrings (HamMed), lumbar part of *M. erector spinae* (ErecLum), thoracic part of *M. erector spinae* (ErecThor), *M. rectus abdominis* (Abdo) and *M. obliquus externus abdominis* (Obli). After a five-minute general warm-up, the subjects performed standardised MVC tasks [18]. Directly thereafter, the subjects received standardised instructions (Table 1) and performed randomized the following types of BE:

- BE_h_: Dynamic flexion and extension of the hip while the spine is stabilised in its neutral position.
- BE_s_: Dynamic flexion and extension of the spine (vertebrae by vertebrae) while the hip is stabilised in its neutral position.

**Table 1:** The following instructions were given to the subject to ensure correct execution of the exercises

Instructions
1. Position yourself in the BE construction such that your upper body is in line with your legs.
2. Position both legs or only the left or the right leg in the construction according to the execution form that you are about to perform.
3. Make sure that your heels are positioned well on the platform of the lower part of the construction and that your knees are straight.
4. Slightly abduct your arms and rotate them externally.
5. Perform eight repetitions of the specific execution form:
a. For the BE_h_, the lowest point is reached right before your spine starts to bend.
b. For the BE_s_, the lowest point is reached right before your hip starts to flex.
6. The maximal extension is reached as soon as your upper body is in line with your legs.
7. Pay attention to the breathing pattern: Exhale during the eccentric phase.

These two types were each executed two-legged (2L) and one-legged (1L, left and right). To allow proper averaging, eight repetitions of each execution form were performed, for a total of 48 repetitions per subject. Between each execution form, there was a break of at least 90 seconds. Because there was no additional loading, this test protocol was expected to be in a submaximal range for BE for all participants.

### Data processing & analysis

The kinetic and kinematic data were reconstructed in Vicon Nexus (version 1.8, Oxford Metrics Group, UK), and any further calculations were conducted with Matlab (version 8.3, The MathWorks Inc., Natick, MA, USA).

The joint centres of ankle, knee and hip were determined functionally using the data from the basic motion tasks [15], whereas the joint centre of L4/L5 was defined anatomically based on anthropometric data [19]. The highest value (peak) reached by the middle point between the shoulder markers and its corresponding time point defined the starting and ending point of each repetition. If there was more than one peak between two repetitions, the first peak was taken as the ending point of the former repetition, and the last peak was taken as the starting point of the following repetition. For the first and last repetitions to be included, the height of the shoulder markers had to lie within 10% of the following or the previous repetition, respectively. The repetitions were time-normalised according to the defined starting and ending points.

The joint moments in the knee, hip and back (L4/L5) were calculated using a quasistatic inverse dynamic approach [20] that considered the joint centres, the ground reaction forces [21] and the gender-specific different segment masses [22]. The resulting joint moments were normalised to the subject’s body mass. Joint angles were based on a least squares fit of redundant skin marker clouds [23] and a helical axis approach [24], and the orthogonal anatomically defined joint coordinate systems [15] were defined. The RoMs for the hip, knee and different parts of the back were calculated, as were the lumbar and thoracic spine curvature and range of curvature [15].

The means and standard deviations over all 8 repetitions of a particular execution form and all subjects were calculated for the RoMs [°] of the middle to upper back (RoM_mub_), lower to middle back (RoM_lmb_), pelvis to lower back (RoM_plb_), hip (RoM_h_) and knee (RoM_k_). For the lumbar and thoracic curvature [1/m], the starting (C_l,s_, C_t,s_) and reversal points (C_l,rp_, C_t,rp_) of the movement and the maximal joint moments [Nm/kg] of back at L4/L5 (M_b,max_), hip (M_h,max_) and knee (M_k,max_) were calculated. Positive values corresponded to flexion angles, and positive moments corresponded to external flexion moments.

EMG data processing involved zero compensation, rectification, a third-order Butterworth (10-500 Hz) and a moving average filter (49 frames) [adapted from: [25]. The following parameters were chosen as the threshold: (20% of the maximal peak), threshold for the on/off-pattern (10% MVC), minimal sub period duration “on” (25 ms) and minimal sub period duration “off” (13 ms). Muscles reaching values higher than three times the MVC for a specific trial were excluded from that trial. The means of the processed EMG signals over all cycles of each subject were then calculated separately for the concentric and eccentric phase of each muscle (mean curve parameters). Furthermore, a Fast-Fourier-Transformation (FFT) was performed to calculate the median frequency both for each repetition and for the whole trial. To evaluate fatigue during each trial and during the whole session, the mean frequency of the first repetition or trial was set to zero, and the deviation of the median frequency of each repetition or trial from this initial value was calculated.

### Statistical analysis

All statistical analyses were conducted using IBM SPSS Statistics (version 22, SPSS AG, Zurich, Switzerland). The normal distribution was visually evaluated using Q-Q-Plots and tested with Kolmogorov-Smirnov-Tests for each parameter. A linear mixed model (significance: p < 0.05) was used for statistical analysis. Significant differences of all parameters between BE_h_ and BE_s_ and among 1L and 2L within BE_h_ and BE_s_ were examined and adjusted using Bonferroni correction within each statistical test.

## Results

In general, no differences between left and right 1L were found. Therefore, they were analysed together. Additionally, the FFT analysis did not show any fatigue between the different trials, which indicated that the breaks were sufficiently long to fully recover between the different execution types. The kinematic and kinetic results for back, hip and knee and the muscular activities are presented below.

### Back

#### Kinematics

For all RoMs (back, hip and knee), significant differences between BE_h_ and BE_s_ were found. Whereas higher RoMs for BE_s_ acted in the middle and upper back, higher values were found in the lower back during BE_h_ compared to BE_s_. (Tables 2 and 3).

**Table 2:** Ranges of motions (RoMs) and maximal external joint moments. RoM [°] of middle to upper back (RoM_mub_), lower to middle back (RoM_lmb_), pelvis to lower back (RoM_plb_), hip (RoM_h_) and knee (RoM_k_), lumbar and thoracic curvature [1/m] at starting (C_l,s_, C_t,s_) and reversal point (C_l,rp_, C_t,rp_) as well as maximal joint moments [Nm/kg] of back (M_b,max_), hip (M_h,max_) and knee (M_k,max_) in the sagittal plane for the different types (BE_h_, BE_s_) and versions (1L, 2L) of BE were given

		RoM_mub_	RoM_lmb_	RoM_plb_	RoM_h_	RoM_k_	C_l,s_	C_l,rp_	C_t,s_	C_t,rp_	M_b,max_	M_h,max_	M_k,max_
		[°]	[°]	[°]	[°]	[°]	[1/m]	[1/m]	[1/m]	[1/m]	[Nm/kg]	[Nm/kg]	[Nm/kg]
BE_h_	1L	8.9 ± 3.7	14.8 ± 6.0	16.6 ± 5.0	29.6 ± 7.4	5.3 ± 2.9	5.6 ± 2.1	-0.1 ± 1.1	2.4 ± 0.7	1.8 ± 0.8	1 ± 0.2	1.1 ± 0.2	-0.8 ± 0.2
	2L	10 ± 4.5	12.5 ± 5.1	17.4 ± 5.0	36.3 ± 7.9	7.4 ± 3.6	5.2 ± 2.0	0 ± 1.2	2.6 ± 0.6	1.8 ± 0.8	1.1 ± 0.2	0.8 ± 0.1	-0.5 ± 0.1
BE_s_	1L	16.6 ± 4.2	36 ± 9.3	11.7 ± 7.3	14.1 ± 9.9	2.9 ± 2.3	5.7 ± 2.2	0 ± 1.9	2 ± 0.7	3.3 ± 0.6	0.9 ± 0.2	0.9 ± 0.2	-0.6 ± 0.2
	2L	14.7 ± 3.2	35.9 ± 10.1	13.8 ± 8.6	15.5 ± 11.7	3.5 ± 3.4	5.4 ± 2.1	-0.6 ± 1.5	2.2 ± 0.7	3.3 ± 0.6	1 ± 0.2	0.7 ± 0.1	-0.4 ± 0.1

**Table 3:** The *p*-values for differences in the mean range of motion, curvature and maximal joint moments in the sagittal plane within and between types and versions

	RoM_mub_	RoM_lmb_	RoM_plb_	RoM_h_	RoM_k_	C_l,s_	C_l,rp_	C_t,s_	C_t,rp_	M_b,max_	M_h,max_	M_k,max_
BE_h_: 1L ↔ 2L	0.201	0.126	0.574	0.001	0.000	0.026	0.907	0.054	0.442	0.651	0.000	0.000
BE_s_: 1L ↔ 2L	0.036	0.942	0.141	0.452	0.153	0.114	0.057	0.001	0.717	0.343	0.000	0.000
1L: BE_h_ ↔ BE_s_	0.000	0.000	0.000	0.000	0.000	0.733	0.693	0.000	0.000	0.000	0.000	0.000
2L: BE_h_ ↔ BE_s_	0.000	0.000	0.033	0.000	0.000	0.413	0.139	0.000	0.000	0.000	0.000	0.001

**Fig. 2:**
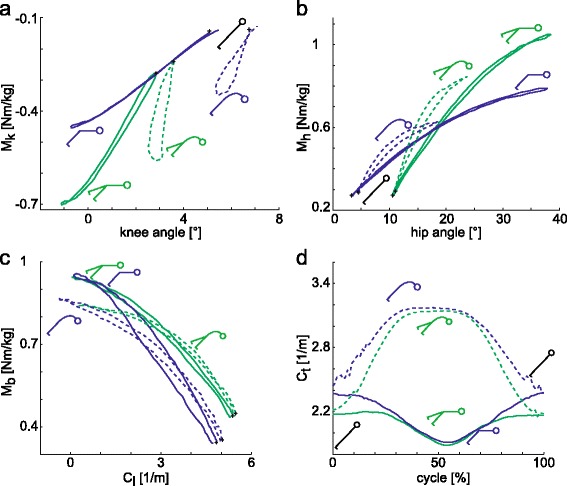
Loading conditions: Moments (positive for external flexion moment) as a function of joint angle averaged over all repetitions and all subject plots are plotted for all BE exercise types (solid: BE_h_, dashed: BE_s_; *blue*: 2L, *green*: 1L; *: starting point) (**a**): Normalised knee moment in the sagittal plane [Nm/kg] as a function of the corresponding knee flexion angle [°]. **b**: Normalised hip moment in the sagittal plane [Nm/kg] as a function of the corresponding hip flexion angle [°]. **c**: Normalised back moment at the level L4/L5 in the sagittal plane [Nm/kg] as a function of the corresponding lumbar curvature [1/m]. **d**: Normalised thoracic curvature in the sagittal plane [1/m] for a cycle [%]

The RoM of C_l_ was comparable for BE_h_ and BE_s_ (Fig. 2c and Tables 2). The curvature in the thoracic region (C_t_) proceeded differently between BE_h_ and BE_s_ during a cycle, which led to significant different starting and reversal points of C_t_ (Fig. 2d, Tables 2 and 3). Moreover, for BE_s_, C_t,s_ was larger for 2L compared to 1L.

#### Kinetics

M_b,max_ was 0.1 Nm/kg greater for BE_h_ than for BE_s_ for all versions and occurred at the reversal point (Fig. 2c, Tables 2 and 3).

### Hip

#### Kinematics

RoM_h_ was twice as high for BE_h_ than for BE_s_ for all versions. Additionally, a shifted starting point for 1L executions could be identified, which led to a smaller RoM_h_ for 1L compared to 2L during BE_h_ (Fig. 2b; Tables 2 and 3).

#### Kinetics

For all versions, M_h,max_ was approximately 0.2 Nm/kg higher for BE_h_ than for BE_s_; for both types, it was approximately 0.3 Nm/kg higher for 1L compared to 2L. M_h,max_ occurred at the reversal point for all execution forms (Fig. 2b, Tables 2 and 3).

### Knee

#### Kinematics

The participants fully extended their knees during BE_h_, whereas during BE_s_, they kept their knees in a slightly bent position. This difference techniques resulted in a greater RoM_k_ for BE_h_. The 1L executions already started with more extended legs compared to 2L, which caused a higher RoM_k_ for 2L executions during BE_h_ (Fig. 2a, Tables 2 and 3).

#### Kinetics

M_k,max_ occurred at the reversal point and was more than 0.1 Nm/kg higher for BE_h_ than BE_s_ for all versions and higher for 1L than for 2L for both types (Fig. 2a, Tables 2 and 3).

### Muscular activity

#### Eccentric phase

GlutMax and GlutMed showed higher activities for the trained leg of 1L executions compared to 2L executions for both types of BE. HamLat was more active for BE_s_ than BE_h_ for 2L. HamMed for BE_h_ and ErecLum For BE_s_ were more active for 1L than 2L, whereas ErecThor showed a higher activity for BE_h_ compared to BE_s_. Abdo and Obli were more active for BE_s_ than BE_h_, however, the activity level for these muscles was very low (Tables 4 and 5).

**Table 4:** Mean curve parameters of *M. gluteus maximus* (GlutMax), *M. gluteus medius* (GlutMed), lateral hamstrings (HamLat), medial hamstrings (HamMed), lumbar part of *M. erector spinae* (ErecLum), thoracic part of *M. erector spinae* (ErecThor), *M. rectus abdominis* (Abdo) and *M. obliquus abdominis* (Obli) for the eccentric and concentric phase of BE

			GlutMax [% MVC]	GlutMed [% MVC]	HamLat [% MVC]	HamMed [% MVC]	ErecLum [% MVC]	ErecThor [% MVC]	Abdo [% MVC]	Obli [% MVC]
Eccentric	BE_h_	1L	17.4 ± 9.9	10.1 ± 5.2	11.9 ± 5.3	12.8 ± 9.7	13.6 ± 4.7	9.3 ± 5.5	2.5 ± 4	3.7 ± 4.4
		2L	12.7 ± 7.0	7.2 ± 4.3	10.5 ± 5.4	9.6 ± 7.5	12.3 ± 4.6	9.8 ± 7.0	2 ± 2.2	3.8 ± 4.2
	BE_s_	1L	17.9 ± 11.0	11.6 ± 6.0	13.8 ± 6.2	14.1 ± 11.2	13.7 ± 6.0	5.6 ± 4.8	3.2 ± 3.4	5.2 ± 5.5
		2L	13.5 ± 7.6	8.2 ± 4.1	14.4 ± 6.5	12.2 ± 8.8	11.8 ± 6.2	4.5 ± 2.4	3.4 ± 3.3	5.1 ± 5.7
Concentric	BE_h_	1L	22.7 ± 12.7	13.5 ± 6.9	16.1 ± 7.1	16.4 ± 11.6	22.8 ± 8.2	9.7 ± 6.0	2.7 ± 3.9	4.6 ± 5.3
		2L	16.5 ± 8.5	9.3 ± 6.0	16 ± 7.0	13.3 ± 10.0	18.9 ± 7.6	9.7 ± 7.7	2.1 ± 2.3	4.3 ± 4.6
	BE_s_	1L	20 ± 12.1	13 ± 7.8	13.8 ± 6.8	13.6 ± 9.7	21.1 ± 9.4	7.6 ± 6.1	2.8 ± 3.3	5.1 ± 4.8
		2L	15 ± 8.6	8.9 ± 5.6	14.4 ± 6.5	12 ± 8.3	17.6 ± 7.8	6.4 ± 4.6	2.9 ± 3.3	4.7 ± 4.9

**Table 5:** The *p*-values for muscle activity within and between types and versions

		GlutMax	GlutMed	HamLat	HamMed	ErecLum	ErecThor	Abdo	Obli
Eccentric	BE_h_: 1L ↔ 2L	0.002	0.001	0.184	0.045	0.153	0.798	0.076	0.978
	BE_s_: 1L ↔ 2L	0.004	0.000	0.504	0.232	0.006	0.193	0.493	0.829
	1L: BE_h_ ↔ BE_s_	0.763	0.098	0.082	0.389	0.451	0.000	0.045	0.039
	2L: BE_h_ ↔ BE_s_	0.579	0.231	0.000	0.093	0.538	0.000	0.000	0.066
Concentric	BE_h_: 1L ↔ 2L	0.001	0.000	0.919	0.068	0.002	0.435	0.044	0.629
	BE_s_: 1L ↔ 2L	0.006	0.000	0.593	0.327	0.001	0.132	0.789	0.530
	1L: BE_h_ ↔ BE_s_	0.123	0.626	0.046	0.092	0.420	0.012	0.480	0.430
	2L: BE_h_ ↔ BE_s_	0.421	0.818	0.171	0.403	0.265	0.001	0.003	0.523

#### Concentric phase

Similarly, GlutMax, GlutMed and ErecLum showed higher activities for the trained leg of 1L compared to 2L. HamLat was more active in BE_h_ during 1L execution, whereas ErecThor showed a higher activity for BE_h_ compared to BE_s_ for all types (Tables 4 and 5).

## Discussion

The aim of this study was to study the influence of different BE execution forms on the spine, hip and knee RoMs, external joint moments and on the muscle activity. In total, 16 subjects performing six different versions of BE were measured and analysed.

### Back

#### Kinematics

Due to the different exercise specifications, higher RoM_mub_, RoM_lmb_ and C_t,rp_ and smaller RoM_plb_ were expected for BE_s_ compared to BE_h_ and could be analysed (Tables 2 and 3). It is remarkable that a similar lumbar spine flexion was found for BE_h_ compared to BE_s_. However, whereas a thoracic spine flexion was analysed during BE_s_, a spine extension was observed during BE_h_ from the starting to the reversal point (Fig. 2c and d). This difference resulted in an opposed movement for the dorsal spine muscles in BE_h_. This opposed movement in the spine during the eccentric part of BE_h_ might affect the dorsal back muscles. ErecLum becomes stretched and experiences an eccentric force, whereas ErecThor contracts in a concentric manner.

The RoMs in the lumbar and thoracic spine of non-fatigued BE reported by Larivière and co-workers [7] were within the values of BE_h_ and BE_s_ of this study. This finding supports the assumption based on the FFT analysis of the EMG data that the BE in the present study were performed in a non-fatigued state. With fatigue, Larivière and co-workers [7] observed RoMs in the lumbar spine that were closer to those of this study’s BE_h_, whereas the values of the RoMs in the thoracic spine were closer to those of the BE_s_ (Table 2). Although the participants tried to start in the exact same position for all execution forms, the significant differences in C_t,s_, especially between BE_h_ and BE_s_, indicated that this was not entirely the case (Tables 2 and 3). Although BE_s_ involved an isolated spine flexion and BE_h_ a stabilised spine, the RoM in C_l_ seemed to be comparable between BE_h_ and BE_s_, which indicates that it was not possible to completely isolate one movement.

#### Kinetics

BE_h_ resulted in a slightly higher M_b,max_ (Fig. 2c, Tables 2 and 3). A higher moment in the back is assumed with a greater exerted strain on the lumbar back muscles, which probably led to a larger training effect on those specific muscles. This difference could be explained by a bigger lever arm of the segmental mass of the upper body in BE_h_. For comparison, the back moments estimated for L5/S1 by Plamondon and co-workers [26] were normalised with a mean body weight (BW, 1.42 Nm/kg). The smaller values for L4/L5 (0.94-1.05 Nm/kg) found in this study can be argued as followed. First, a less horizontal leg position causes a smaller external torque [6] and second, a smaller lever arm due to L4/L5 being more cranial compared to L5/S1 is presented. Furthermore, Plamondon and co-workers [26] described that the maximal back moment occurred at or near a horizontal trunk position, which agrees with this study's M_b,max_ occurring at the reversal point along with the smallest C_l_ (Fig. 2c).

Comparing found back moments to other back strength exercises, such as deadlifts and goodmornings, the maximal moment in the back, M_b,max_, is two to three times smaller during BE [maximal back moment = 2.75–2.81 Nm/kg with 25% extra barbell load for deadlifts and goodmornings; 27].

### Hip

#### Kinematics

Because of different exercise specifications, RoM_h_ was expected to be higher for BE_h_ compared to BE_s_. This assumption was confirmed by the results (Fig. 2b, Tables 2 and 3). Larivière and co-workers [7] achieved a RoM_h_ of 13°, which was in accordance with this study’s BE_s_ values. The smaller RoM_h_ for 1L compared to 2L (Fig. 2b) could be explained by the inability of the participants to fully extend their hip in the starting position of 1L executions due to a lack of stabilising capacity.

#### Kinetics

Similar to the kinetic results in the lower back, the maximal moment in the hip, M_h,max_, was slightly higher for BE_h_ compared to BE_s_ (Fig. 2b, Tables 2 and 3), which presumably resulted in a higher training effect for the dorsal hip muscles during BE_h_. Additionally, a significantly higher M_h,max_ and, thus, probably a better training effect were achieved for 1L compared to 2L for both types of BE (Fig. 2b, Tables 2 and 3).

Comparing to other strength exercises, M_h,max_ during 2L BE was similar to squats without any extra load [27, 28] and was approximately half for 2L and two thirds for 1L of the load reached by deadlifts and goodmornings with 25% BW extra barbell load [29]. The latter is quite astonishing, considering that the BE were performed without any extra load. For comparison with this study's results, the maximal hip moment for 45° BE calculated by Contreras and co-workers [6] was normalised with BW (5.43 Nm/kg). Because they used an external weight of approximately 50% BW, it is reasonable that their value is approximately five times larger than what the present study measured for M_h,max_.

### Knee

#### Kinematics

Unsurprisingly, a small RoM_k_ was expected and confirmed in this study because the BE were performed with straight legs (Fig. 2a, Tables 2 and 3). Similar RoM_k_ values were obtained for the straight leg exercise goodmornings [29]. The RoM_k_ is significantly higher for BE_h_, and the starting positions differ between BE_h_ and BE_s_ (Fig. 2a, Tables 2 and 3). To increase the RoM_h_ for BE_h_, participants might have tilted their pelvis forward, which could have promoted knee extension and thus increased the RoM_k_. However, compared to other strength exercises, the RoM_k_ is still extremely small. In the starting position of 1L, the knee might have been more extended than in the starting position of 2L due to stabilising reasons and higher moments (Fig. 2a, Tables 2 and 3).

#### Kinetics

For the knee joint not being directly involved in the movement, high M_k,max_ were obtained, especially for BE_h_ and 1L executions. It is important to note that M_k,max_ is an extension moment and is thus supporting the isometric contraction of the hamstrings and providing a training effect for these muscles. In addition to the positive effect on the hamstrings, the extension moment might exert forces on the passive structures in the knee, which must be considered in patients with current or past knee injuries. As far as we know, no one has ever looked at the kinematics and kinetics in the knee during BE although this exercise might shift the quadriceps/hamstring ratio towards hamstring as part of an Anterior cruciate ligament (ACL) prevention program. The knee extension moments obtained for goodmornings were slightly higher, probably due to the extra barbell load of 25% BW [29].

### Muscular activity

Because BE are a dynamic exercise, the EMG results must be treated and interpreted with caution due to skin artefacts. This caution is especially important for the comparison between BE_h_ and BE_s_, which are different movements.

In general, all muscles were more active during the concentric phase than during the eccentric phase (Tables 4 and 5). For the concentric phase, the activity levels for gluteal muscles and hamstrings agree with the literature [1]. The activity level for lumbar and thoracic dorsal back muscles are considerably lower (lumbar: 39-56% MVC, thoracic: 43% MVC; [1]). For gluteal muscles and hamstrings, no significant differences between BE_h_ and BE_s_ were found, which agrees with the results of Mayer and co-workers [8], who did not find any influence of lumbar posture on these muscles. Moreover, they also observed that an accentuated lumbar lordosis increased the activity of the lumbar extensors, whereas the present study found, for ErecLum, no significant differences between BE_h_ and BE_s_. However, due to the different MVC measurement settings, electrode placements, starting positions and exercise executions, a direct comparison between different studies is difficult. The only study looking at the muscle activities in the eccentric phase of the BE exercise was a study by De Ridder and co-workers [13], who considered BE performed in a horizontal position and with an extra load of 60% 1RM. Due to the extra load, a comparison of the values between their study and the present study is not reasonable.

As expected, the ventral trunk muscles were almost inactive (2-5% MVC), which agrees with the results of Callaghan and co-workers [11]. The high standard deviations (see Table 4) suggest that some participants needed to activate those muscles more than others to stabilise the trunk.

### Comparison of types BE_h_ and BE_s_

Apart from the obvious differences in RoM_mub_, RoM_lmb_ and RoM_h_, there were other relevant differences between BE_h_ and BE_s_. BE_h_ showed higher external moments in hip, knee and lower back with higher or similar RoMs (Fig. 2a-c, Tables 2 and 3). This finding suggests a higher training effect for BE_h_ for dorsal back and hip muscles and for hamstrings. However, the above-mentioned opposed movement in the spine for BE_h_ must be considered.

### Comparison of versions 1L and 2L

The 1L version showed higher external moments in the hip and knee with smaller or similar RoMs (Fig. 2a-c, Tables 2 and 3). Because strength training requires high moments with high RoMs, we are unsure whether 1L provides a greater training effect for the dorsal hip muscles and hamstrings than 2L. These results suggest that the version should be chosen according to the specific requirements, i.e., whether high RoM or high external moments are desired.

## Conclusions

In strength training, high moments with wide RoMs in the trained joint are desired in order to minimise the load on other parts of the body. To strengthen the hip and lower back, BE_h_ seem to be more efficient due to higher moments with higher or similar RoMs in the hip and lower back. According to Callaghan and co-workers [11], disk deformation, ligament and spinal loading can be reduced if BE exercises are performed with neutral lordosis. Therefore, BE_h_ are not only more efficient for training but also healthier. However, athletes and patients must be aware of the opposed movement in the lumbar and thoracic spine when performing BE_h_.

Due to the extension moment in the knee and the flexion moment in the hip, 1L BE in particular, provide an effective training to strengthen the dorsal part of the limb in a specific RoM, especially the hamstrings and glutes. However, patients with knee deficits must be aware of the high external moments in the knee in 1L BE. In the future, kinetic and kinematic analysis combined with subject specific musculoskeletal modelling allow to quantify the individual loading conditions during strength training exercises.

## Abbreviations

(M_k,max_): Maximal joint moments of the knee [Nm/kg]
1 L: One leg
2 L: Two legs
Abdo: *M. Rectus abdominis*
ACL: Anterior cruciate ligament
BE: Back extension
BE_h_: Back extension dynamic hip
BE_s_: Back extension dynamic spine
BW: Body weight
C: Thoracic curvature [1/m]
C_l_: Lumbar curvature [1/m]
C_l,rp_: Lumbar curvature at the reversal point [1/m]
C_l,s_: Lumbar curvature at the start [1/m]
C_t,rp_: Thoracic curvature at the reversal points [1/m]
C_t,s_: Thoracic curvature at the start [1/m]
EMG: Electromyography
ErecLum: Lumbar part of *M. erector spinae*
ErecThor: Thoracic part of *M. erector spinae*
FFT: Fast-Fourier-Transformation
GlutMax: *M. Gluteus maximus*
GlutMed: *M. Gluteus medius*
HamLat: Lateral hamstrings
HamMed: Medial hamstrings
L: Leg
L4/L5: Between lumbar vertebra 4 and 5
M_b,max_: Maximal joint moments of the back at L4/L5 [Nm/kg]
M_h,max_: Maximal joint moments of the hip [Nm/kg]
MVC: Maximum voluntary contraction
Obli: *M. Obliquus externus abdominis*
RoM: Range of motion [°]
RoM_h_: RoM in the hip [°]
RoM_k_: RoM in the knee [°]
RoM_lmb_: RoM lower to middle back [°]
RoM_mub_: RoM middle to upper back [°]
RoM_plb_: RoM pelvis to lower back [°]
RoMs: Ranges of motion [°]
